# Reversal Learning in Humans and Gerbils: Dynamic Control Network Facilitates Learning

**DOI:** 10.3389/fnins.2016.00535

**Published:** 2016-11-17

**Authors:** Christian Jarvers, Tobias Brosch, André Brechmann, Marie L. Woldeit, Andreas L. Schulz, Frank W. Ohl, Marcel Lommerzheim, Heiko Neumann

**Affiliations:** ^1^Faculty of Engineering, Computer Science and Psychology, Institute of Neural Information Processing, Ulm UniversityUlm, Germany; ^2^Special Lab Non-Invasive Brain Imaging, Leibniz Institute for NeurobiologyMagdeburg, Germany; ^3^Department Systems Physiology, Leibniz Institute for NeurobiologyMagdeburg, Germany

**Keywords:** reversal learning, expert networks, recurrent neural networks, reinforcement learning, adaptive resonance theory, stability-plasticity

## Abstract

Biologically plausible modeling of behavioral reinforcement learning tasks has seen great improvements over the past decades. Less work has been dedicated to tasks involving contingency reversals, i.e., tasks in which the original behavioral goal is reversed one or multiple times. The ability to adjust to such reversals is a key element of behavioral flexibility. Here, we investigate the neural mechanisms underlying contingency-reversal tasks. We first conduct experiments with humans and gerbils to demonstrate memory effects, including multiple reversals in which subjects (humans and animals) show a faster learning rate when a previously learned contingency re-appears. Motivated by recurrent mechanisms of learning and memory for object categories, we propose a network architecture which involves reinforcement learning to steer an orienting system that monitors the success in reward acquisition. We suggest that a model sensory system provides feature representations which are further processed by category-related subnetworks which constitute a neural analog of expert networks. Categories are selected dynamically in a competitive field and predict the expected reward. Learning occurs in sequentialized phases to selectively focus the weight adaptation to synapses in the hierarchical network and modulate their weight changes by a global modulator signal. The orienting subsystem itself learns to bias the competition in the presence of continuous monotonic reward accumulation. In case of sudden changes in the discrepancy of predicted and acquired reward the activated motor category can be switched. We suggest that this subsystem is composed of a hierarchically organized network of dis-inhibitory mechanisms, dubbed a dynamic control network (DCN), which resembles components of the basal ganglia. The DCN selectively activates an expert network, corresponding to the current behavioral strategy. The trace of the accumulated reward is monitored such that large sudden deviations from the monotonicity of its evolution trigger a reset after which another expert subnetwork can be activated—if it has already been established before—or new categories can be recruited and associated with novel behavioral patterns.

## 1. Introduction

Agents, be they biological or technical systems, which operate and behave in changing environments require cognitive and behavioral flexibility. Such behavioral flexibility is essential for survival and represents a unique component of intelligence, in particular the ability to switch flexibly between different behaviors and to reuse strategies that have been established previously and used selectively in different circumstances (Kinoshita et al., 2008; Kangas and Bergman, 2014). The neural correlates of behavioral flexibility of humans and animals are often measured using *reversal* learning experiments (Pubols, 1957; Xue et al., 2013). In such experimental settings, a subject first learns to map a complex input stimulus of a certain category onto a behavioral response. Subsequently, in a second phase, the subject learns to overrule the previously established stimulus-response mapping based on changing reinforcement contingencies. Now the input stimulus is mapped onto a behavioral response that opposes the previously established output category. During *serial reversal learning* the agent and its cognitive system are exposed to multiple changes of contingencies. After acquisition of a reversed contingency, the mapping rules reverse again such that the initial mapping conditions are re-established.

Humans and animals are able to learn such serial reversals and their efficiency gradually increases with multiple switches (Robbins and Roberts, 2007). This increased efficiency of performance in serial reversal tasks cannot be explained by learning mechanisms which are solely based on the initially formed associations of complex stimulus features to categorical responses, since this leads to decreased efficiency due to interference effects from the previously learned contingencies (Pubols, 1957; Clayton, 1962; Gossette and Inman, 1966; Feldman, 1968; Gossette and Hood, 1968; Kulig and Calhoun, 1972; Garner et al., 1996; Bathellier et al., 2013; Kangas and Bergman, 2014).

In order to model serial reversal learning tasks some authors (Graybiel, 1998; Frank and Badre, 2012) proposed the inclusion of expert networks in which independent reinforcement learners, e.g., standard neural networks, are trained for each sub-task or contingency condition to perform complex cognitive functions. These experts are then selectively activated by a gating network (Jacobs et al., 1991a,b; Graybiel, 1998) or in a hierarchically organized structure (Frank and Badre, 2012). Such a processing scheme suggests that a rich set of behavioral “templates” is established to build a repertoire for generating appropriate behavioral responses given the specific contextual conditions. Thus, one expert network would acquire the stimulus-to-motor mapping that is optimal in the first experimental phase. After the first reversal, another expert takes over and acquires the new mapping, preserving the learned weight in the first expert. Thus, when a second reversal takes place, the original motor mapping is still present in expert one and can be applied quickly.

In order to incorporate this idea in a full, biologically plausible reinforcement learning architecture, the relationship between the different experts has to be clarified. Cortical anatomy suggests that they are not completely autonomous modules which can be switched on or off depending on certain sensory or behavioral state conditions. Instead, a conjoint sensory processing stream is accessible and read out by different brain areas, e.g., the ventrolateral prefrontal cortex and the lateral orbitofrontal cortex, to perform more complex cognitive functions (Fellows and Farah, 2003; Clark et al., 2004; Hornak et al., 2004; Boulougouris et al., 2007; Kinoshita et al., 2008; Rygula et al., 2010). In addition, distinct areas, e.g., nucleus basalis, are capable of modulating cortical processing such that a more continuous activation and deactivation of functionalities emerges (instead of the activation of mutually exclusive programs) (Roberts et al., 1990). We address this issue by extending a learning algorithm for recurrent neural networks (Brosch et al., 2015) to a hierarchical network architecture, in which multiple expert sub-networks receive input from the same sensory driven basis network and are modulated by a common dynamic control mechanism.

Secondly, a biologically plausible mechanism has to be proposed for selecting among the expert networks. This mechanism has to explain how a single expert is selected and how the responses of the other expert networks are suppressed. Many of the selection mechanisms proposed in previous work have not been formulated in a biologically plausible manner (e.g., Jacobs et al., 1991b). Here, we propose a dynamic control network that steers the selection of the experts using a competitive release-from-inhibition mechanism. Such a scheme of neural dis-inhibition structurally resembles a subdivision of the basal ganglia (Gurney et al., 2001a).

Finally, one needs to explain how the same expert network can be selected repeatedly while the reward contingencies remain constant, but a different network can be selected once contingencies reverse. We propose an adaptive biasing mechanism for the dynamic control network, which resembles a stabilized Hebbian outstar learning scheme (Grossberg, 1980) which is further extended by a monitoring of the reward prediction error. This approach enables the network to select expert networks solely based on the prediction error. In contrast, many other proposals rely on additional contextual cues (Jacobs et al., 1991a; Frank and Badre, 2012; Collins and Frank, 2013) or task instructions (Niv et al., 2015) to switch between strategies.

We use this hierarchical, dynamically controlled reinforcement learning architecture to model behavioral data from humans and gerbils in serial reversal learning tasks. The extended architecture is compared to a monolithic architecture without dynamic control. We find that both human participants and gerbils learn faster after the second compared to the first reversal and that only the extended network with dynamic control replicates such behavior.

## 2. Materials and methods

### 2.1. Experimental setups and protocols for behavioral studies—animal and human

#### Human experiments

##### Participants

21 subjects participated in the experiment that took place inside a 3 Tesla MR scanner (11 female, 10 male, age range between 18 and 34 years, all right handed, with normal hearing). All subjects gave written informed consent to the study, which was approved by the ethics committee of the University of Magdeburg, Germany.

##### Experimental design

240 frequency-modulated (FM) tones served as experimental stimuli. The tones differed in duration (short, 400 ms, vs. long, 800 ms), intensity (low intensity, 76–81 dB, vs. high intensity, 86–91 dB), frequency range (low, 500–831 Hz, vs. high, 1630–2639 Hz), direction of frequency modulation (rising vs. falling) and speed of frequency modulation (slow, 0.25 octaves/s, vs. fast, 0.5 octaves/s). The relevant stimulus properties for the categorization task were duration and modulation direction, resulting in four basic tone categories: short/rising, short/falling, long/rising, and long/falling. For each participant, one of these categories constituted the target sounds (25%), while the other three categories served as non-targets (75%).

Participants were instructed to listen to each sound while looking at a fixation cross and to select target category sounds via a left button press with the index finger and to reject sounds via a right button press with their middle finger. Immediately after each of the 240 trials subjects received verbal feedback depending on whether they made the right choice. Participants were not told that contingencies might change. They were informed that the experiment would last about 30 min and that two resting periods of 20 s duration each would be introduced indicating that they finished the first and the second third of the experiment, respectively.

As feedback stimuli, four positive utterances (ja, “yes”; richtig, “right”; ja, richtig, “yes, right”; stimmt, “correct”) and four negative utterances (nein, “no”; falsch, “wrong”; nein, falsch, “no, wrong”; stimmt nicht, “not correct”) as well as one time-out utterance (zu spät, “too late”) were employed, all spoken in standard German and with a motivational intonation taken from the evaluated prosodic corpus MOTI (Wolff and Brechmann, 2012; see also Wolff and Brechmann, 2015). After 80 trials, a pause of 20 s was introduced and from the next trial on the contingencies were reversed such that the target stimulus required a push of the right instead of the left button. After 160 trials and another pause of 20 s a re-reversal was introduced such that the initial assignment of target sounds and button 1 was valid again. Participants were divided into learners and non-learners based on their individual performance averaged across 20 trials per block. A subject is considered to be a *learner* if he/she has obtained 80% correct responses in at least one block of the initial learning and the reversal learning phase. Otherwise, the subject is assigned to the group of *non-learners*. For the first and second reversal phase, the time was determined for each participant as the ordinate (blocks of 20 trials) of the first linear intersection between the criterion level (80%) and performance rate.

#### Animal experiments

All procedures were performed in accordance with the European Communities Council Directive of November 24, 1986 (86/609/EEC), and according to the German guidelines for the care and use of animals in laboratory research. Experiments were approved by the Ethics Committee of the state Saxony-Anhalt.

Male Mongolian gerbils (*n* = 18) were trained in a two-way active avoidance paradigm (Ohl et al., 1999) in a shuttlebox. Specifically, the animal subjects were trained to discriminate frequency modulation direction of tones (2–4 kHz and 4–2 kHz) with their behavioral responses. In each experimental session, one of these stimulus types serves as Go and the other as NoGo stimulus. During Go trials, shuttling to the other compartment within 6 *s* after stimulus onset was scored as a *hit*, while longer latencies led to a *miss* score. In this case a mild foot shock was delivered via the grid floor of the shuttlebox. During NoGo trials animals were free to stay in the current compartment or to shuttle and no shock was delivered. Shuttling behavior during NoGo tone presentation was counted as a *false alarm*. In both tone conditions the tone was switched off after shuttling behavior. During Go trials tones were turned off either after successful shuttling or with the delivery of the foot shock (*miss* condition). Daily sessions consisted of 96 trials presented in a pseudo-randomized order of Go and NoGo trials. Discrimination performance was monitored on a daily basis by assessing psychophysical detection performance values *d*′ for hit and false alarm rates. If the animal subject reached the criterion of three sequential sessions with *d*′ values ≥ 1 it advanced to the next training phase with reversed stimulus contingencies.

For the analysis of the behavioral performance animals were classified as *learners* if they reached a *hit*-rate above 70% in the initial training and the two sequential contingency changes. *Non-learners* did not reach the 70% level and were not considered in the subsequent time-to-criterion analysis. For the time-to-criterion analysis, “sessions to criterion” were determined as the ordinate (session) of the first linear intersection between criterion level and hit rate. Since we observed a high variability in single-subject learning rates, an individual criterion level for each gerbil-subject was calculated as 80% of the median hit rate in all three training phases of that particular animal. Note that the animals did not receive a shock in case of shuttling during the NoGo tone (the former Go tone after stimulus contingencies reversal). The next reversal was introduced only once the gerbils received a discrimination performance of *d*′ ≥ 1 in two out of three consecutive sessions. An increase in shuttling to the former NoGo stimulus could only be observed if shuttling on NoGo trials was relatively low prior to the contingency reversal.

The foot shock acts in a two-way active avoidance task (as in this described Go/NoGo discrimination task) as a negative reinforcer. A negative reinforcer leads to an increase of a behavior, in this case the shuttling behavior in order to actively avoid the foot shock. Already Mowrer (1956) interpreted in his two-factor theory the emergence of the avoidance response to a conditioning stimulus (tone) as a result of release of fear (the CS becomes associated with UCS) after the avoidance response and consequently the termination of the tone. In turn, the release of fear might elicit similar neuronal mechanism as caused by positive reinforcement. Recent measurements of dopamine in the striatum during acquisition of avoidance behavior in a shuttlebox showed an increased release of dopamine during the first successful trials (Dombrowski et al., 2013). Similar release of dopamine has been observed during learning with positive reinforcers (Schultz, 2001). Therefore, we think the shuttlebox active avoidance paradigm is similar to and can be modeled like learning reinforced by reward.

Thus, both in human subjects and animals had to learn a behavioral strategy based on reinforcement and to switch strategies when reward contingencies change. The switch in reward contingencies was not accompanied by an external signal and were only able to be recognized based on a change in reward prediction error. We examined whether participants could switch strategies and reuse previously learned strategies under these conditions. The goal of the model was to replicate this behavior.

### 2.2. Modeling

In this section, we briefly recapitulate the REinforcement LEarning Algorithm for Recurrent Neural Networks (RELEARNN) described in Brosch et al. (2015). For further details about the algorithm, its motivation and biological plausibility, the reader is referred to the previous paper. We outline the network topology and the learning algorithm, and then describe the extension by a dynamic control network. RELEARNN serves as a generic framework for a biologically realistic learning mechanism (Brosch et al., 2015) in which sensory information serves as input to the network and each output unit is associated with a possible action like pressing a button or jumping over a hurdle. RELEARNN has been shown to be a biologically plausible model able to explain behavior as well as electrophysiological recordings in two challenging contour grouping tasks. We reasoned that such a model architecture may serve as a functional building block for modeling the mapping of sensory input to actions in humans and gerbils.

#### RELEARNN: general network topology

The REinforcement LEarning Algorithm for Recurrent Neural Networks (RELEARNN) is a learning algorithm for simultaneous recurrent neural networks, i.e., networks that process static inputs (not time series), aiming to compute the values of actions as a consequence of the sensory input. Model units' dynamics follow an ordinary differential equation, which describes the average membrane potential in a cortical column. Learning is achieved by updates of (synaptic) weights, which are computed after a stable state is reached. The model contains a number of output units, each representing a possible motor action. The magnitude of activity corresponds to the expected reward for the associated action given the current sensory input. In other words, these action values, dubbed *Q*-values (Sutton and Barto, 1998), are encoded by the activity of the output units. The model usually chooses the action with the highest *Q*-value, but it will occasionally also explore other actions to promote learning by using a softmax output function (Roelfsema and van Ooyen, 2005). To find an appropriate balance between biological detail and mathematical tractability we used model units with scalar activation values assuming a rate coded activity pattern as output from a neuron population (hence, we do not consider spiking neurons in our model). The activity of each model unit represents the average activity in a cortical column with mean membrane potential *p* and mean firing rate *g*(*p*). As inputs, the model units receive excitation, inhibition as well as modulatory influences and the units, in turn, can inhibit, excite, or modulate other model units. The role of the modulatory connections is to amplify the influence of excitatory input, but they are unable to drive the units (c.f. Shao and Burkhalter, 1996; Sherman and Guillery, 1998; Larkum et al., 2004; Bonin et al., 2005; Spratling, 2014).

**Figure 1:**
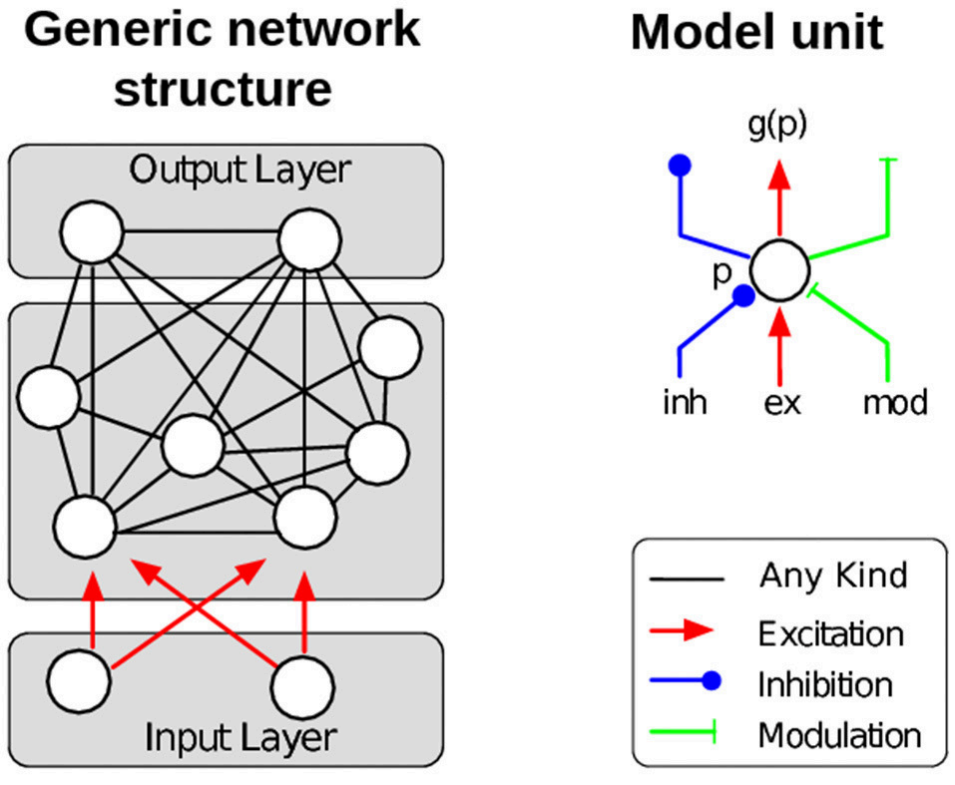
**Left:** Illustration of the general network structure as used in the description of the learning algorithm. Each model unit *n*_*i*_ can excite, inhibit or modulate the activity of any other unit *n*_*j*_ (non-directional black connections). Units $n_{i}^{I}$ of the input layer do not depend on the activity of other units. **Right:** Each model unit corresponds to a cortical column and can be excited, inhibited or modulated by other model units. Redrawn after Brosch et al. (2015).

The membrane potential *p* depends on the excitatory, inhibitory, and modulatory inputs *I*^*ex*^, *I*^*inh*^ and *I*^*mod*^ as follows (Figure 1, right):

(1) $$\begin{array}{ll} \frac{d}{dt}p & =-\alpha p+\left(\beta -p\right)\cdot I^{ex}\cdot \left(1+\gamma I^{mod}\right)-\left(\zeta +p\right)\cdot I^{inh}. \end{array}$$

The decay rate of the activity of model units is controlled by α > 0, the maximal activity by β > 0, the minimal activity by ζ > 0, and the parameter γ > 0 determines the impact of modulatory input. The mean spike rate *r* is calculated as:

(2) $$r=g(p)=\{\begin{array}{ll} a+p, & p\geq 0,\\ a\;\cdot \;\exp(p/a), & p<0. \end{array}$$

We consider a network of *N* dynamically interacting model units with activities **p**_*i*_ receiving excitatory input **I**^*inp*^ (see Figure 1 for the general structure of such a network). Once the input is provided, the activity circulates through the excitatory, inhibitory and modulatory connections until the network activity stabilizes. The overall dynamics are described by the following system of coupled differential equations (similar to Equation 1), but now presented in vector notation).

(3) $$\begin{array}{ll} \frac{d}{dt}\text{p} & =-\alpha \text{p}+\left(\beta -\text{p}\right)\cdot {\text{I}}^{ex}\cdot \left(1+\gamma {\text{I}}^{mod}\right)-\left(\zeta +\text{p}\right)\cdot {\text{I}}^{inh}. \end{array}$$

The excitatory, inhibitory and modulatory inputs **I**^*ex*^, **I**^*inh*^ and **I**^*mod*^ depend on the presynaptic firing rates and the input into the network **I**^*inp*^:

(4) $$\begin{array}{ll} {\text{I}}^{ex} & ={\left({\text{W}}^{ex}\right)}^{T}\cdot g\left(\text{p}\right)+{\left({\text{W}}^{inp}\right)}^{T}\cdot {\text{I}}^{inp}, \end{array}$$

(5) $$\begin{array}{ll} {\text{I}}^{inh/mod} & ={\left({\text{W}}^{inh/mod}\right)}^{T}\cdot g\left(\text{p}\right). \end{array}$$

Here, α, β, γ, ζ ≥ 0 and *g*(·) (applied element-wise) are defined as in Equation (1) and **p**, **I**^*inp*^ ∈ ℝ^*N*^ are column vectors of the activations and inputs of each unit. The positive elements ${\text{W}}_{kl}^{\left(\cdot \right)}\geq 0$ of the weight matrices **W**^(·)^ ∈ ℝ^*N*×*N*^ determine the connection strength from unit *k* to unit *l* and ${\text{W}}_{kl}^{inp}$ determines the excitatory connection strength from feature *k* to input unit *l* (here products of two column vectors like **p** · **I** ∈ ℝ^*N*^ are defined element-wise). When the activity in the network has converged to a stable state, the network chooses one action based on the activation of the output units that encode the action values (*Q*-values). We used the softmax rule to determine the probability *p*_*i*_ of an output unit *i* to win the competition between actions based on their values:

(6) $$\begin{array}{l} p_{i}=\frac{\text{exp}\left({\text{p}}_{i}/\tau \right)}{\sum_{j\in O}\text{exp}\left({\text{p}}_{j}/\tau \right)}, \end{array}$$

where τ is called a temperature parameter (Sutton and Barto, 1998). We did not model here how the softmax action selection process is implemented in the neural network, although this has been addressed in previous work (Nowlan and Sejnowski, 1995). Moreover, the choice of softmax as an action selection rule is not critical. We expect that other action selection mechanisms used in the reinforcement literature (e.g., ε-greedy Sutton and Barto, 1998, or max-Boltzmann Wiering and Schmidthuber, 1997) will give qualitatively similar results.

#### RELEARNN: weight updates

**Figure 2:**
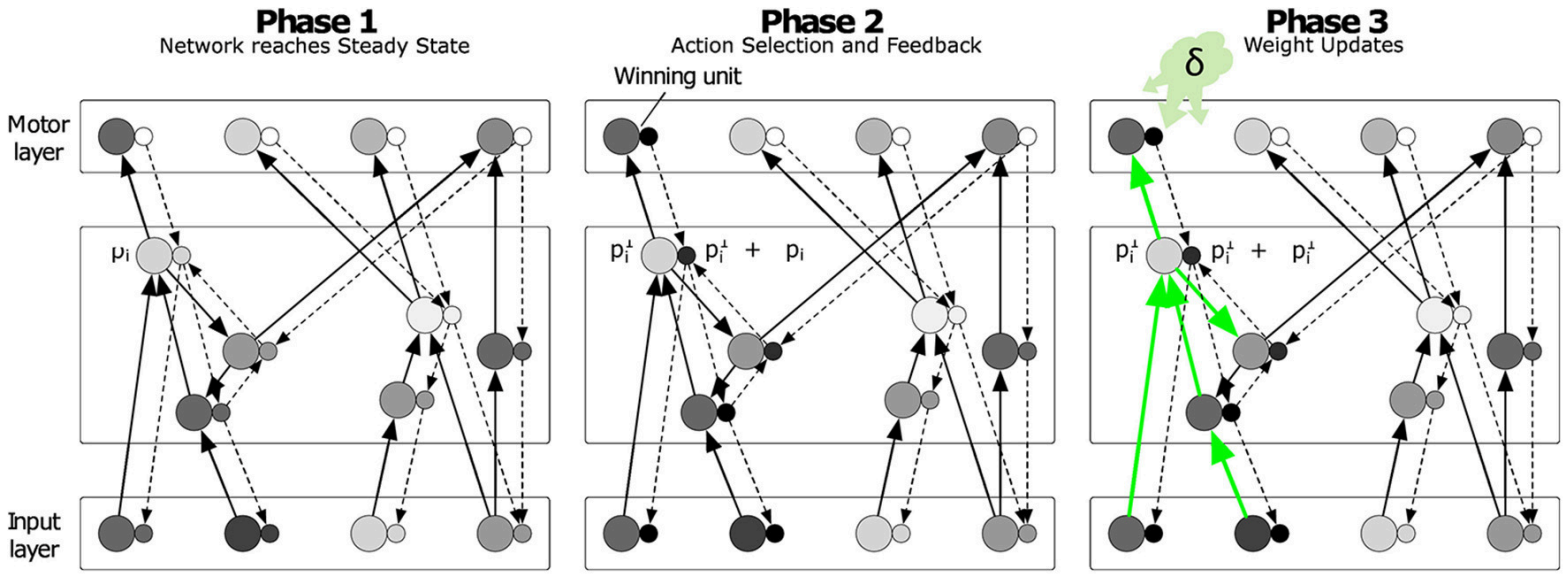
**Illustration of the learning phases**. Each regular unit (large circles) is accompanied by an accessory unit (small circles), which are hypothesized to be situated in the same cortical column. In phase 1, the sensory input leads to a stable state **p**^∞^ of the regular units (note that we only illustrated the excitatory connections in this scheme) and the model represents estimates of the value of all the actions in the output layer. In phase 2, the winning output unit injects extra activity into the accessory network. The strength of the connections of the accessory network is reciprocal to that of the regular network. Accessory units that are paired with a regular unit that has a strong impact on the activity of the winning unit exhibit a strong increase in activity Δ**p** during this phase. In phase 3 the changes in synaptic strength depend on Δ**p** and a neuromodulatory signal that encodes the reward-prediction error δ. Reproduced from Brosch et al. (2015).

The learning algorithm has been derived under constraints of biological plausibility, particularly considering the local mechanisms of synaptic weight adaptation. The model is best described in three phases (cf. Roelfsema and van Ooyen, 2005; Friedrich et al., 2011; Rombouts et al., 2012). Phase one starts in response to the input and ends when the network converges to a stable state **p**^∞^ and stochastically selects action *a* according to Equation (6). In the second phase, the selected output unit *a* causes an action feedback signal (AFB) that propagates through the network through a separate set of units (one per column; small circles in Figure 2) which change their response by Δ**p** during this phase so that their total activity becomes **p**^∞^+Δ**p**. We call the network of units sensitive to the AFB the “accessory network” (see below for details), which is important for the guidance of the process of synaptic plasticity. The strength of the connections between units in the accessory network is similar (or proportional) to the strength of connections between the regular units (larger circles in Figure 2). This reciprocity of regular and accessory connections can emerge during the learning process itself (see also Roelfsema and van Ooyen, 2005). In Brosch et al. (2015) we have shown that the boost in the membrane potential Δ**p**_*l*_ of the accessory unit *l* during the second phase is proportional to the influence of a change in **p**_*l*_ on the activity of the current winning unit ${\text{p}}_{a}^{\infty }$ during the first phase. Therefore, the sign and magnitude of Δ**p**_*l*_ can be used to guide plasticity once learning is initiated in the third phase in response to the reward. The output units of the network aim to represent the expected reward value if their action is chosen in the current sensory state:

(7) $$\begin{array}{ll} Q_{a}={𝔼}_{\pi }\left\{\rho |s,a\right\}. & \end{array}$$

When the network performs action *a*, it receives a reward ϱ and the aim of the learning rule is to adjust the current estimate of *Q*_*a*_, represented by the activity of the winning output unit ${\text{p}}_{a}^{\infty }$. To this aim, the network computes a reward prediction error δ by comparing the outcome of the trial ϱ to the predicted *Q*-value, i.e., a SARSA style prediction error for immediately rewarded tasks (Sutton and Barto, 1998),

(8) $$\begin{array}{ll} \delta & =\varrho -Q_{a}=\varrho -{\text{p}}_{a}^{\infty }. \end{array}$$

In accordance with previous studies of reinforcement learning (Schultz et al., 1997) there is growing evidence that such a reward prediction error is encoded by a non-specific neuromodulatory signal that is globally released into the (sensory) network so that it can influence the plasticity of all synapses (Figure 2, right panel). Candidate mechanisms for such neuromodulatory mechanisms are dopamine neurons (in the ventral tegmental area and substantia nigra; Schultz, 2002; Montague et al., 2004; Schultz, 2007) or acetylcholine (in the basal forebrain and brainstem; Pennartz, 1995; Warburton et al., 2003).

Once the network has received feedback about the chosen action *a*, the learning rule changes the connections of the network in order to decrease the reward prediction error for this action. Plasticity of a specific connection *w*_*kl*_ from unit *k* to unit *l* depends on four factors: (1) the presynaptic activity $g\left({\text{p}}_{k}^{\infty }\right)$, (2) the postsynaptic membrane potential ${\text{p}}_{l}^{\infty }$, (3) the activity of the accessory unit *l* $\Delta {\text{p}}_{l}^{\infty }$, which represents the influence of unit *l* on the activity of *a*, and (4) the reward prediction error δ, leading to the following synaptic learning rule:

(9) $$\begin{array}{ll} \Delta {\text{W}}_{kl} & =\eta \cdot \delta \cdot \Delta {\text{p}}_{l}^{\infty }\cdot f_{l}\left({\text{p}}_{l}^{\infty }\right)\cdot g\left({\text{p}}_{k}^{\infty }\right), \end{array}$$

where η denotes the learning rate. Note that the signals that determine plasticity are all available locally in the cortical column *l* and that Equation (9) implements a form of Hebbian plasticity because it depends on the product of presynaptic activity ${\text{r}}_{k}^{\infty }$ and a function *f*(·) of the postsynaptic activity ${\text{p}}_{l}^{\infty }$. The form of *f*(·) differs between excitatory, inhibitory and modulatory connections projecting to column *l* and is determined by the core equations that cover the dynamics of the cortical columns. We get:

(10) $$\begin{array}{ll} {\text{f}}_{l}^{ex}\left({\text{p}}_{l}^{\infty }\right) & =\left(\beta -{\text{p}}_{l}^{\infty }\right)\cdot \left(1+\gamma \cdot {\left({\text{I}}_{\infty }^{mod}\right)}_{l}\right), \end{array}$$

(11) $$\begin{array}{ll} {\text{f}}_{l}^{mod}\left({\text{p}}_{l}^{\infty }\right) & =\gamma \cdot \left(\beta -{\text{p}}_{l}^{\infty }\right)\cdot {\left(I_{\infty }^{ex}\right)}_{l}, \end{array}$$

(12) $$\begin{array}{ll} {\text{f}}_{l}^{inh}\left({\text{p}}_{l}^{\infty }\right) & =-\left(\zeta +{\text{p}}_{l}^{\infty }\right) \end{array}$$

see Brosch et al., 2015, for further details and for the derivation of this learning rule and its connection to the Almeida-Pineda algorithm for recurrent backpropagation; Almeida, 1987; Pineda, 1987).

This model structure served as the central building block for the learning of connectivity weights to maximize the predicted reward for associated motor activities generated in behavioral experiments.

#### Extended RELEARNN: dynamic control

**Figure 3:**
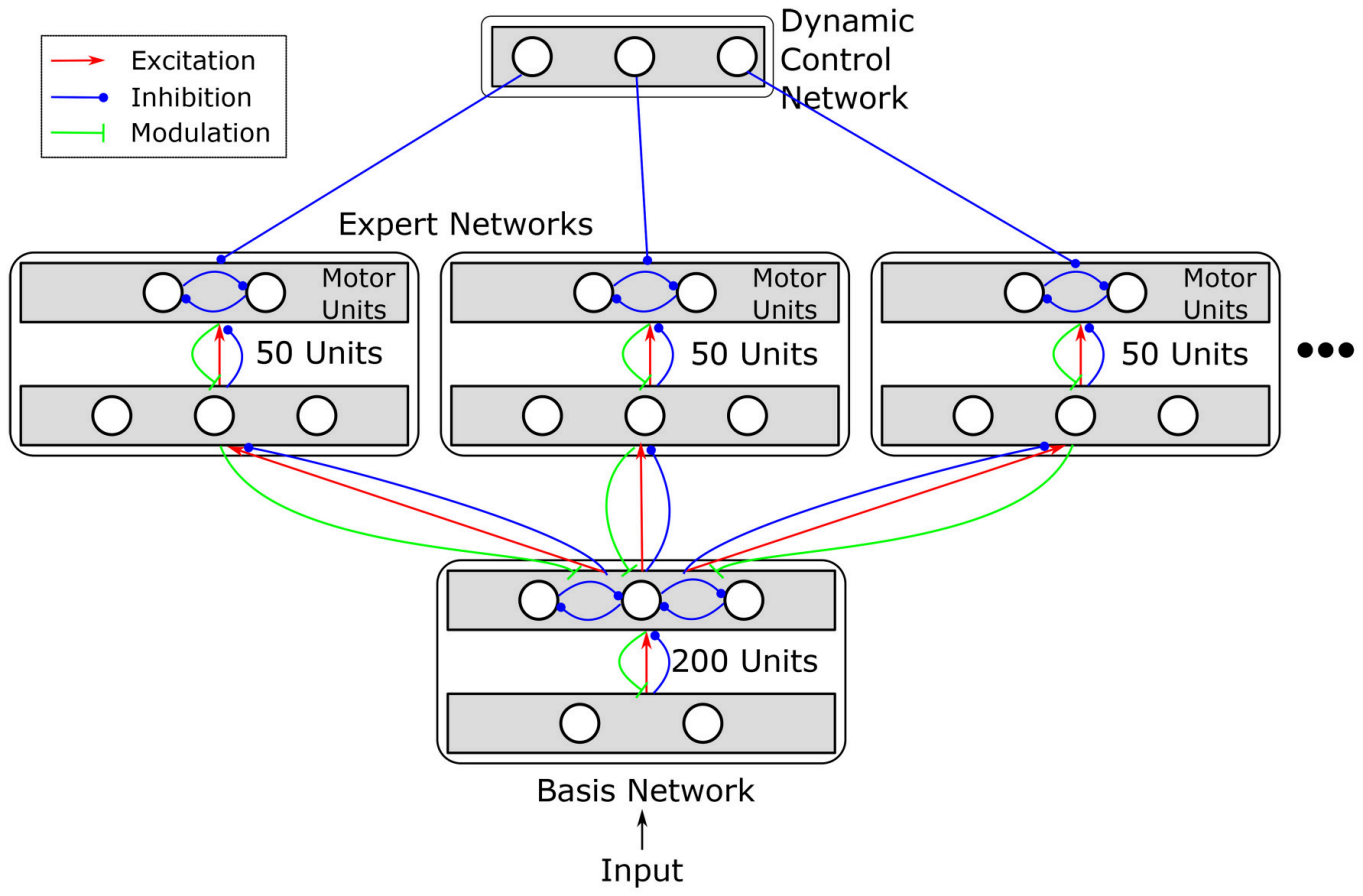
**Structure of the extended network**. Different motor mappings are realized by multiple expert networks, each of which comprises 50 units. All expert networks receive input from the same sensory basis network. Basis network and expert networks together correspond to an original RELEARNN network (see Figure 1) with restricted connectivity. Additionally, a dynamic control network (DCN) inhibits all experts but one. The structure of the DCN is detailed in Figure 4.

In order to model serial reversal learning, we extend the basic RELEARNN network to a hierarchical multi-expert architecture inspired by ideas outlined in e.g., Jacobs et al. (1991a,b); Pennartz (1997); Graybiel (1998). We divided the network into several components organized in a hierarchical manner: a basis network, which receives the input and achieves strategy-independent sensory feature extraction, several expert networks, which learn the sensory-to-motor mapping, and a dynamic control network, which selects among the experts (see Figure 3). Thus, the network solves the problem of serial reversal learning by acquiring several behavioral strategies, optimally one per reward contingency, each of which is instantiated by a different expert network. These subdivisions are established by restricting connectivity within the network. The units in each expert network receive excitatory input from and send modulatory feedback to the basis network, but there are no connections between the experts. Thus, an important property of our network design is that learning not only affects the currently active expert network, consisting of 50 units each, but also the sensory basis network consisting of 200 units^1^.

The basis and expert networks consist of recurrently connected layers. The basis network consists of two layers. Units in both layers can excite/inhibit and receive modulatory feedback from every other unit in the next layer (c.f. Figure 3). Units in the second layer of the basis network engage in a mutual competition which—as any other connection—is subject to learning. The expert networks consist of one association layer and two motor units which are meant to encode the motor programs relevant to the task, i.e., to shuttle or to remain (gerbils task) or to press the left or right button (human experiment). Expert networks can provide modulatory feedback to the last layer of the basis network and receive feedforward excitation/inhibition. Thus, regardless of which expert network currently determines the response, all expert networks can influence the basis network.

**Figure 4:**
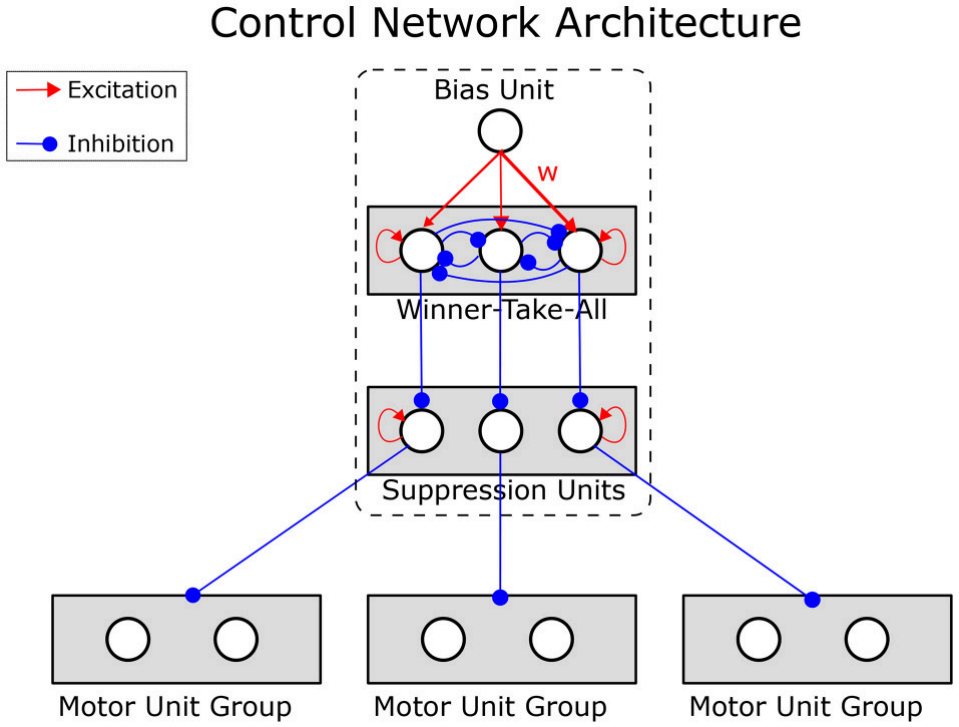
**Structure of the dynamic control network**. Motor units are inhibited by suppression units. One of the suppression units is itself suppressed by the winning unit of a winner-take-all circuit, releasing the motor units of a single expert network from inhibition. Which of the winner-take-all units wins is determined by the connections from a tonic bias unit. The strengths (**w**) of these connections from the bias unit to the winner-take-all units are learned from the history of the reward prediction error, enabling the network to learn the strategy selection. The dashed box corresponds to the Dynamic Control Network box in Figure 3.

The motor units of each expert network predict the reward values of the associated actions. Therefore, interference effects could easily arise, impairing the ability of each expert to learn independently of the others. The dynamic control network prevents this by inhibiting the output units of all expert networks but one. This is achieved by a competitive field that controls the motor units by release from inhibition (see Figure 4).

The units in the competitive field implement a winner-take-all mechanism (WTA). The network can select expert networks strategically by learning appropriate weights for the connections from the bias to the winner-take-all units. The weights **w** are learned according to the rule in Equation (13).

(13) $$\begin{array}{ll} \Delta \text{w}={\text{p}}_{B}^{\infty }\cdot \left({\text{p}}_{WTA}^{\infty }-\text{w}\right)+\sigma \cdot \delta \cdot \left(c-\text{w}\right)+\delta \cdot \nu & \end{array}$$

where ${\text{p}}_{B}^{\infty }$ is the activation of the bias unit at equilibrium, ${\text{p}}_{WTA}^{\infty }$ are the activations of the WTA units at equilibrium, δ is the prediction error, ν is a noise term and the constants *c* and σ are model parameters, which control how quickly the model switches experts in response to large prediction errors. Thus, while the prediction error is low, the learning rule behaves like outstar learning (Grossberg, 1980) and learns to predict the outcome of the WTA. This leads to a repeated selection of the same expert network. If, on the other hand, the prediction error rises, the weights are attracted toward the constant *c* and the selection becomes more noisy, enabling other units to win the competition and activating different expert networks. Consequently, the learned bias weights act like working memory, as they enable the system to remember to which strategy it currently adheres. The bias weights are reset after a higher than usual prediction error occurs. This corresponds to a reorientation of this working memory resource and bears resemblance to the orienting system in adaptive resonance theory (Carpenter and Grossberg, 1993, see Section 4.3).

#### Simulation parameters

Experiments utilized frequency modulated auditory stimuli differing in duration, intensity, frequency range, direction of frequency modulation and speed of frequency modulation. Stimuli were encoded using a population code which in our case consisted of two units for each stimulus dimension. Thus, stimuli in our simulations were encoded by ten binary units. We used the same stimulus configuration as in the human experiments in which a combination of two stimulus dimensions was task relevant. Thus, the model had to learn to ignore the other three stimulus dimensions.

We simulated two different networks, one with multiple (three) experts and a dynamic control network and one network with a single expert and without dynamic control. As in Figure 3, 200 units were used in the sensory basis network and 50 units were used per expert network.

Model parameters were fitted empirically to reproduce the first learning phase of the human data. We note, however, that results did not critically depend on the precise parameter choice. We set the rewards in successful trials to ϱ = 0.4 and to ϱ = 0 in erroneous trials. The learning rate was set to η = 0.2, a momentum term α = 0.9 was used to speed up learning, the softmax temperature (c.f. Equationn 6) was set to τ_*h*_ = 0.15 and the parameters of the neuron model (Equation 1) were set to α = β = δ = 1 and γ = 4. Connection weights were initialized by uniformly drawing numbers from [0, 0.2] except for connections to the output layer which were drawn from [0, 0.3] to speed up learning and the modulatory connections which were drawn from [0, 0.1]. The inhibitory weights in the dynamic control network were initialized to 4, the maximum weight permitted in the simulation. The excitatory bias weights were initialized in the range [0.8, 1.2]. The parameters of the control network learning were set to σ = 0.9, *c* = 0.2 and ν was uniformly drawn from [−0.01, 0.01].

Each learning phase (the initial learning phase (Initial), the phase after the first reversal (REV01) and after the second reversal (REV02)) consisted of a fixed number of 160 stimulus presentations.

## 3. Results

### 3.1. Experimental results

We conducted serial reversal experiments with humans and gerbils (compare Section 2.1). In both experiments, the performance criterion was reached faster in the second than in the first reversal.

#### Human reversal learning

**Figure 5:**
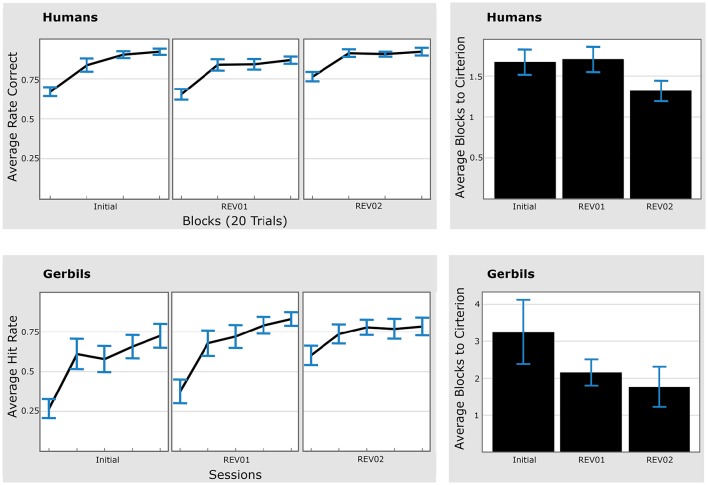
**Humans and gerbils are faster in the second reversal phase as compared to the first reversal phase (REV01/REV02). Left:** Average hit rates (for gerbils) and average rate of correct responses (for humans). **Right:** Time to criterion. **Top:** Human data. **Bottom:** gerbil data. Average hit rates reached asymptotic performance faster during each reversal phase. Error bars represent standard errors of the mean. Initial: initial learning, REV01: first reversal, REV02: second reversal.

We recruited 21 participants, of which 16 (76%) reached the 80% correct response criterion in all three phases, including the 2nd reversal phase (Figure 5, left panel). Three out of 21 participants (14%) were not successful in the initial learning phase. Another 2 subjects were not successful during the first reversal phase. The results of the remaining participants are shown in a composite arrangement in Figure 5 (left panel).

Pairwise comparisons between the first blocks of the *2nd (REV02)* and *1st (REV01) reversal phase* were evaluated and revealed *significantly better performance* during the 2nd reversal block (*p* = 0.01, 2-sided paired Wilcoxon test). This improved performance was also reflected in the time to criterion (Figure 5, right panel) which was reached significantly faster in the 2nd compared to the 1st reversal phase (*p* = 0.03, 2-sided paired Wilcoxon test).

#### Animal reversal learning

Fifteen animal subjects out of 18 that were trained in the serial reversal task were classified as “learners” since they reached hit rates over 70% during the discrimination and sequential contingency reversals. For each animal an individual criterion was calculated as 80% median of the overall attained hit rate from all training sessions. The average individual criterion level was at 64% hit rates with 95% confidence intervals [59%, 70%]. One animal out of the 15 animals reaching the 70% hit rate criterion was classified as an outlier, as its time to criterion value in the REV01 phase was larger than 4 times the standard deviation of the group. Note that including this animal did not affect the Wilcoxon statistic significantly, but lead to an even larger averaged time to criterion for REV01.

Similar to humans, gerbils showed a significantly shorter time-to-criterion when the second reversal was compared to the first reversal phase (*p* = 0.023, 2-sided paired Wilcoxon test; Figure 5, *bottom right*). The improvement was also reflected by the significant difference (*p* = 0.002, 2-sided paired Wilcoxon test) between the hit rates of the first session in the respective phase (REV02 vs. REV01). Different learning speeds in the initial learning phase are discussed in Section 4.6. In addition, animals showed a significantly shorter time-to-criterion when the initial discrimination learning was compared to the first contingency reversal (*p* = 0.035, 2-sided paired Wilcoxon test).

### 3.2. Simulation results

To obtain a quantitative comparison to the experimental data, simulation runs of the model were similarly analyzed for their performance. Simulation runs were classified as having learned the task (learner) if they responded correctly in over 70% of the trials in each of the three phases. Time to criterion was assessed as the ordinate of the first linear intersection between the criterion level (80%) and the rate of correct responses (over blocks of 40 trials).

**Figure 6:**
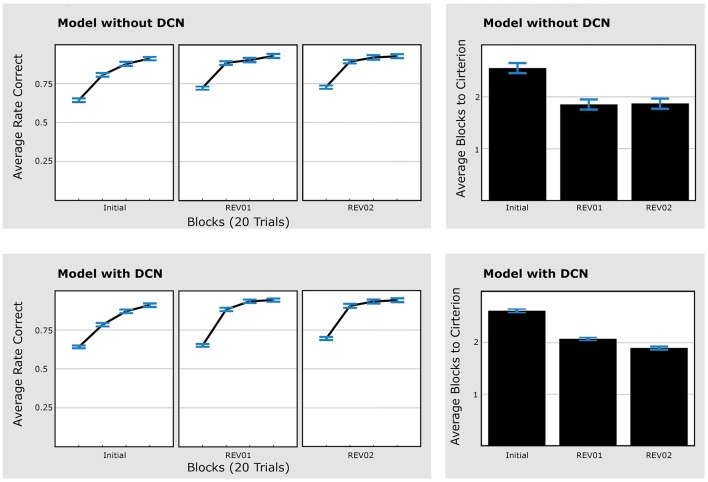
**Model with DCN explains faster learning speed in second reversal (REV02) compared to REV01**. Performance data of simulation runs without DCN (*upper panels*) and simulation runs with DCN (*lower panels*). The upper left panels show the average rate of correct responses of the 53 simulation runs that reached 70% performance in all three learning phases (“learners”). The upper right panel shows the time to criterion. Unlike humans and gerbils the model without DCN did not improve from the first to the second reversal phase. From the model with DCN, 73 simulation runs reached criterion to classify as “learner” (*lower panels*). In accordance with the experimental data within these simulations the criterion was reached significantly faster, comparing the first reversal phase to the second one (*lower right panel*). Error bars represent standard errors of the mean. Initial: initial categorization learning, REV01: first reversal, REV02: second reversal.

Out of 100 simulation runs of the model version *without* DCN, 53 were classified as “learners.” There was a significant decrease in the required number of blocks to reach the criterion from the initial phase to REV01 [paired *t*-test, *t*_(52)_ = 5.78, *p* < 0.001], but no change from REV01 to REV02 [paired *t*-test, *t*_(52)_ = 0.14, *p* = 0.891; Figure 6, *upper panels*), which is *not* consistent with the experimental data. Using the model version *with* DCN, 73 simulation runs passed the learning criterion. Like in the model without DCN, there was a significant decrease in the number of blocks needed to reach a criterion of 80% performance [paired *t*-test, *t*_(72)_ = 6.26, *p* < 0.001] comparing the initial learning phase to REV01. In accordance with experimental data there was also a significant decrease from REV01 to REV02 for this model [paired *t*-test, *t*_(72)_ = 2.11, *p* < 0.038; Figure 6, *lower panels*].

Consequently, only the model with the DCN reproduces the experimental result of a faster time to criterion for REV02 compared to REV01. This is in line with the theoretical considerations outlined before. In contrast to a network with a single expert, the model with DCN and multiple expert networks can re-employ the first expert network instead of degrading existing and establishing new connections.

## 4. Discussion

### 4.1. Main findings

In this study, we propose a dynamic control mechanism which can selectively activate specific expert networks while silencing others for independent strategy acquisition during serial reinforcement learning. We used animal and human experimental paradigms together with computational modeling and show that this extended model explains the behavioral data better than a standard reinforcement learning scheme when the agent is forced to change its behavioral strategy multiple times. The learning mechanism of the DCN exploits the sudden rise of the reward prediction error following immediately after a reversal. Such a situation is different from those conditions being investigated in, e.g., operant conditioning with respect to different homeostatic drives (Harlow et al., 1950; Grossberg, 1971; Chang and Gaudiano, 1998; Keramati and Gutkin, 2011) and also unlike context changes reflecting a significant variation in the environment in which otherwise the same behavioral operation is demanded (Bouton, 2002; Bouton and Todd, 2014). Rather, the agent needs to register a significant variation in the evaluative conditions within the environment.

The main contributions of the study are three-fold. First, we demonstrated that the reinforcement learning rule for establishing perceptual grouping mechanisms presented in Brosch et al. (2015) is also suitable for behavioral learning. Second, we present experimental findings from animal as well as human studies using a behavioral task that induces multiple strategy reversals for the agent. The results showed that after the strategies have been successfully established, the previously trained strategy after a second reversal can be adopted faster than the new strategy after the first reversal. The subjects learned to transfer previous experiences to a new reversal phase. Third, based on these experimental findings we observed that a neural network trained with standard reinforcement learning mechanisms is not sufficient to account for the faster strategy adoption after the second reversal. In such a scheme, a sudden change in the received reward leads to a large difference in predicted and received reward which in turn leads to a complete unlearning and re-establishing of connections related to the motor-mapping. We extended the basic model architecture by adjoining a control mechanism which dynamically selects one of multiple expert sub-networks, which determines the current action. This *dynamic control network* (DCN) qualitatively accounts for the reported experimental findings.

### 4.2. Relation to previous experimental findings and models of learning action repertoires

Our proposal for an extended network builds on previous work on reinforcement learning and multi-expert architectures. For example, Jacobs et al. (1991b) suggested a supervised learning scheme based on feedforward processing that establishes a system of experts, each specialized to solve a specific subtask in an overall complex interpretation task. While each expert receives the input simultaneously to generate different outputs specific for the respective subtask, a gating network learns in parallel to control the selection of each expert. Graybiel (1998) argued that cortico-striatal mapping in habit and action learning may rely on such a multiple expert architecture, identifying the striatum as a possible substrate of the experts, which can be modulated by dopamine signals.

Similarly, Frank and Badre (2012) proposed an elaborate hierarchical reinforcement learning model that implements a Bayesian mixture-of-experts approach. This model is able to prioritize information, and thereby instantiate different stimulus-response mappings, depending on context. For example, the model could learn to respond to stimulus orientation in the presence of one colored box, while it would respond to stimulus shape in the presence of a different colored box.

Another multiple-experts architecture is the multiple model-based reinforcement learning algorithm (Doya et al., 2002). However, the experts are not coordinated by a single gating network. Instead, each learner is paired with a world model, which predicts the environmental state (e.g., the movement of prey). The influence of each expert as well as its propensity to learn are scaled by the correctness of the prediction of the associated world model. Only the experts associated with a correct prediction learn and decide the action outcome, making partitioning of the problem space possible. This architecture excels at control tasks, in which the correct action depends on an observable state of the environment, which can be predicted by the world models (Doya et al., 2002). However, in settings like the reversal learning tasks in this study, where the only external signal that carries information about the task state is the reward itself, the world models do not have sufficient information to select appropriate experts. A similar concern applies to other model-based reinforcement learning models (for a review see Doll et al., 2012): they can model serial reversal learning, provided that sufficient sensory information is available to learn to predict the world state.

Our network shares several properties with these previous proposals in that the expert networks learn distinct behavioral strategies and compete for action selection. This competition is resolved by an additional (sub-)network that selectively gates or inhibits the experts. However, there are important differences between our framework and the cited models. For example, unlike the model of Jacobs et al. (1991b), we here utilize a reinforcement scheme for learning in which the output selection is based on a stochastic selection mechanism. Also, unlike these previous models our reinforcement learning adapts not only the expert network but also the hierarchically organized basis (sensory) network for feature detection. As indicated in Figure 2 the reward-based learning signal is distributed globally to minimize the reward prediction error for those representations that contribute to the action category. This leads to a continuous adaptation—although with small amounts—of the feature extraction mechanisms when different experts are activated. In other words, the sensory input processing is adapted to improve the function of the currently active expert network.

The most crucial difference to previous work is the mechanism of the dynamic control network. In other proposals, the supervisory network typically receives a specific contextual input that indicates the current task state. Jacobs et al. (1991a) provide a special input signal that indicates the current task state and in Jacobs et al. (1991b) the gating network received the same input as the expert networks. Similarly, in Frank and Badre (2012) one stimulus dimension explicitly encodes the current task state. In contrast, the dynamic control network in the current proposal learns solely based on the reward prediction error and can, therefore, also learn successfully in situations in which no external cue indicates which strategy is currently required. This is the case in reversal learning experiments, in which the stimuli do not provide cues about the current task.

Another model in which experts can be switched in the absence of contextual information is outlined in Wilson and Niv (2012); Niv et al. (2015). The authors describe two models, one of which uses optimal Bayesian inference to estimate the current rewarding dimension and feature, taking into account that the reward contingencies may have switched. The other model is sub-optimal but more efficient. It selectively attends to a specific stimulus dimension for some time and learns the reward contingencies for this dimension. Additionally, the model is able to shift attention to another stimulus dimension using Bayesian hypothesis testing. Thus, these models can explain reversal learning in which the relevant stimulus dimension changes, such that, for example, the participants first have to react to low-frequency sound and later to low-intensity sounds. However, in paradigms where the relevant stimulus remains the same and only the correct action changes, such as the human experiments described in this study, these models do not predict faster learning after the second reversal. Notably, the authors tie their model to specific brain areas, including prefrontal cortex and the basal ganglia.

Another Bayesian model (Donoso et al., 2014) monitors the reliability of several alternative strategies simultaneously and acts according to the one that is currently most reliable. If a contingency change renders all current strategies unreliable, then an exploratory stage is initiated in order to learn a new strategy. Crucially, both the strategy learning and switching are guided by the prediction error, as in our model and in the model of Niv et al. (2015). The authors showed that the processes of their model correlate with activations in the prefrontal cortex and the basal ganglia (Donoso et al., 2014).

Recently, it has been suggested by O'Reilly and Pauli (2010) that representations in prefrontal cortex are dynamically gated to determine when representations are updated or preserved. The subnetwork responsible for this gating process consists of the interconnected basal ganglia, frontal cortex, and thalamus. It is suggested to adaptively control action repertoires to build a flexible sequence of actions as part of procedural knowledge (Graybiel, 1995). The modulation of such action templates is suggested to evaluate the reinforcement of repertoires. These principles are the basis for more recent investigations which demonstrate that striatal neuron activity is involved in establishing (encoding) and adaptation (recoding) of actions and activities, considered as procedural memories (Barnes et al., 2005). Interestingly, such learned action repertoires, considered as experts with focused tuning properties, could be reactivated after they had been selectively shut off (compare also Mink, 1996, for an earlier overview). Such a switching behavior necessitates an internal monitoring mechanism to evaluate the success of a specific program. In our network architecture, such a monitoring function is conducted by the DCN which selectively inhibits specific expert networks or action categories. We suggest that such a monitoring function can be learned using only the reward acquired by the agent. If the prediction error for the reward is consistently high, the current strategy is not appropriate and a switch occurs due to a shift in biasing top-down input to the competitive field of the DCN. This resembles the functionality of the orienting system which was suggested as part of adaptive resonance theory (Grossberg, 1980, 1987) that uses a vigilance mechanism that is triggered by a top-down signal and encodes the expected matching input. In the case of a mismatch the input and top-down attentional expectation are dissimilar and trigger a reset signal that shuts off the currently active category cell representation. In the scheme proposed here, we suggest that the selection signal is not generated by an evaluation of the signal characteristic but rather by the obtained reward over an extended temporal period of the agent's interaction with its environment. Taking findings obtained from long-term recordings of sensorimotor striatum into account (Barnes et al., 2005), our results suggest that cortico-ganglial circuits of learners reliably encode changes in task representations while these correlates would be missing in subjects that fail to solve the task.

### 4.3. Action category selection by a dynamic control network

A key motivation of the proposed network architecture and its computations is derived from layered bidirectionally coupled neural mechanisms in adaptive resonance theory (ART), originally proposed by Grossberg (1980). ART principles have originally been proposed to explain mechanisms of stable online category learning in neural systems. Later the framework was operationalized to realize an online associative memory mechanism for unsupervised learning of categories in object recognition tasks (see Carpenter, 1989). A core element of this framework is the specification of mechanisms which allow a neural system to automatically acquire new knowledge (by assigning new category nodes to novel input) and to adapt existing nodes to input variations. Input and category layers are connected bidrectionally. While feedforward connections convey input feature representations, feedback signals in ART architectures help solve the stability-plasticity dilemma. Top-down signals carry predictions from category nodes of the expected input feature representation. As long as the feature representation matches the top-down prediction, learning by weight adaptation takes place for the active category. However, if representation and prediction produce a mismatch, the currently active category is switched off to allow another category node (with better matching representation) to become active or to recruit a new category node. This match-adapt and mismatch-reset principle keeps those previously acquired representations stable and prevents catastrophic forgetting when existing category representations are overshadowed by new input.

Building upon this key framework, we propose a similar mechanism for the establishment and dynamic selection of expert networks. Similar to the inhibition of the active category after a mismatch in ART, the current behavioral or motor category is suppressed in response to a high prediction error. This prevents catastrophic forgetting after reward contingency changes. We suggest here that categories in our system are defined by small subnetworks (of a few hundred model neurons) which receive input feature representations from sensory processing. These subnetworks can be considered as expert networks of the kind as proposed by Graybiel (1995). In our conceptual framework we suggest that a category node (at the output layer of the model network) represents the interface to a competitive layer of categories to be selected. Here, categories encode predicted reward values (instead of object categories) which are compared to the achieved reward. The reward indicates the appropriateness of the (motor) action selected by the system. The comparison between predicted reward, viz activity of the selected category node, and the acquired reward can be considered as an orienting system with vigilance as in ART systems, which is activated when new and unexpected events occur and induce mismatches between bottom-up input and top-down expectation. Here we propose that such an orienting system is realized in a cascaded subsystem to steer a reinforcement learning mechanism. The learning itself is achieved in three separate phases, namely the activation and selection of the output category, the tagging of those synaptic weights that were involved in the overall network computation to calculate the predicted reward at the output, and the global adaptation signal (difference between predicted and received reward) effective at the tagged synapses. In ART this orienting system is suggested to include the non-specific thalamus and the hippocampal system (Carpenter and Grossberg, 1993). For the monitoring of the reward accumulation for sequences of behavioral choices we argue that a different complementary subsystem evaluates the success of the reward acquisition. A monotonic trace of rewards indicates ongoing success of behavioral choices. In case of strong deviations from such a trace, conditions might have changed and/or selections might no longer be appropriate and should therefore be adapted. The current motor category with its associated expert network is switched off and another subnetwork is selected. This orienting subsystem has been dubbed *Dynamic Control Network* (DCN).

Unlike the vigilance level of the orienting subsystem in ART, the DCN itself is subject to learning, resulting in a bias of competition between motor categories. In a nutshell, the competition is biased to further establish a routine behavioral selection when the predicted and accumulated reward fulfill a matching condition. The neural correlates of this biasing mechanism remain to be investigated. In the current model, we implemented the bias using a single model unit, which projects to the competitive field of the DCN with weights learned using an outstar-like learning scheme (Grossberg, 1980), which fulfills two purposes: it stabilizes the competitive field while the reward prediction error remains low and resets the competition once the prediction error rises. A neuronal population that implements this mechanism would be expected to show activity during the reset phase when the prediction error is high. Furthermore, its activation strength should predict strategy reversals. Whether such a population can be found remains to be investigated in future work.

The DCN itself is hierarchically organized to implement a dis-inhibitory network that releases a category from inhibition. Such organization resembles the cascade of different nuclei in the basal ganglia system. In particular, dis-inhibitory chains of striato-nigral (Pars reticularis) as well as striato-pallidal (Pars interna) projections to the ventrolateral thalamus are involved in dopaminergic control of the cortico-striatal loop (Gurney et al., 2001a). We suggest that the proposed networks, responsible for the selection of proper behavioral motor templates, might be selectively activated and concerted by the action of subnetworks of the basal ganglia. Furthermore, the learning of sensory representations as well as motor action templates is based on a temporally and spatially distributed system of modulatory influence that controls the adaptation of connection weights in the sensory as well as the behavioral control systems.

An alternative approach to implement dynamic control over behavioral strategies has been taken by Maniadakis et al. (2012). They used genetic algorithms to instantiate networks that can activate one of several fixed actions, retain the current strategy in a working-memory-like manner, and switch between strategies in response to punishment. Due to their evolutionary approach, learning and control are separated into different phases. Thus, their model applies to a somewhat different paradigm than the one explored here, in which the agent knows the possible reward contingencies and does not need to learn them on the fly. Interestingly, Maniadakis et al. (2012) observed that a hierarchically structured network is much more likely to learn switching strategies correctly.

### 4.4. Differences in reinforcement paradigms

We show that the proposed architecture can qualitatively capture the strategy-switching behavior and reestablishment of earlier strategies observed in humans and gerbils. Nevertheless, some differences between the paradigms for human and gerbil experiments have to be taken into account when interpreting the results. The main differences pertain to the nature of the reward (positive and negative reinforcer), the reward distribution for non-target sounds, and the task instructions.

In the gerbil task, negative reinforcers (foot shocks) were administered, whereas positive and negative reinforcers (feedback utterances) were used for human participants. As was argued above (Section 2.1), we assume that the same learning mechanisms underlie positive and negative reinforcement, and treat the release of fear (Mowrer, 1956) after a successful avoidance response as a reward. Thus, here we only model learning by reinforcement. In the current formulation, the model does not learn from punishment (i.e., negative reward values) alone, as the motor units predict the reward directly using their mean spike rate, which is strictly positive. In order to model learning from punishment without the assumption of a negative reinforcement effect, a further transduction step would be required to map the mean spike rate onto a range that includes negative values as well.

In addition to the absence of positive reinforcement in the gerbil paradigm, the distribution of rewards also differed from the human experiments: whereas human participants received negative feedback if they responded incorrectly to a non-target stimulus, the gerbils did not receive any reinforcer during the NoGo trials. Consequently, shuttling on every trial irrespective of the stimulus would constitute an optimal strategy for this task as the foot shock would be avoided on every trial. This holds true under the assumption that there is no behavioral bias against shuttling. Such bias, for example, translates to a decreased reward value after shuttling, rendering indiscriminate switching suboptimal. The experimental evidence shows that gerbils did not shuttle on every trial but reached the discrimination performance criterion between Go- and NoGo-tone, indicating that such a bias exists.

Finally, the experiments differed in the instructions which the participants received. Human participants were instructed to respond to target category tones with button presses. They were not told about the reversals or the target category, but the instructions provided an initial understanding about the task that the gerbils and the model lacked. This is reflected in the faster learning speeds of gerbils and the model after the first reversal, compared to the initial learning phase. During the initial learning, the gerbils and the model had to learn that there was a meaningful distinction between target and non-target tones. This knowledge could be transfered to the second learning phase such that, e.g., in the model the trained sensory basis network builds separable feature representations that support the decision-making.

Furthermore, the human participants may have used their abstract knowledge about the task structure and relied on higher cognitive functions. For example, after experiencing a contingency reversal in the second phase of the experiment they may have predicted a further reversal in the third phase and used cognitive control to inhibit the current strategy (for possible mechanisms, see Mansouri et al., 2012). Such additional influences on the behavioral strategies are not considered in the current form of the model but may be explored in future work.

### 4.5. Role of the sensory basis network

An important feature of our model is the sensory basis network, which provides inputs to all expert networks. It corresponds to a sensory processing stream, e.g., in auditory cortex, which can acquire sensory representations that are relevant for the task, but not specific to the learned strategies. Notably, the sensory basis network also learns based on the back-propagated reward prediction error. This is in line with experimental findings indicating that early auditory cortical areas are activated in response to dopaminergic reward signals (Puschmann et al., 2012) and show learning-dependent plastic changes (Weis et al., 2013).

**Figure 7:**
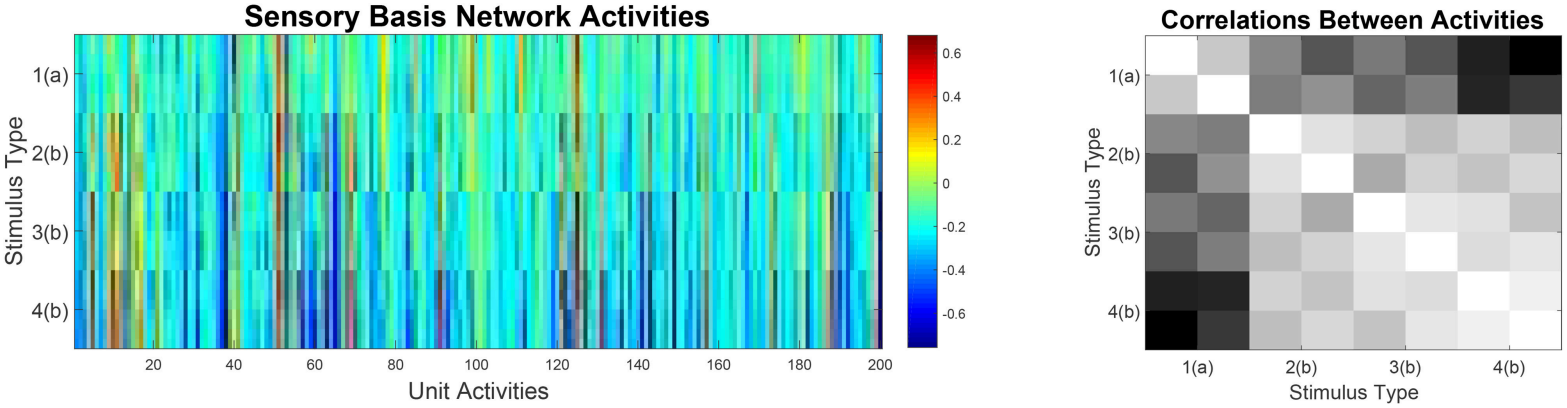
**Left:** Activities in the trained sensory basis network when stimulated with all 32 possible stimuli. Notably, stimuli that only differ along dimensions which are not task relevant induce highly similar activities. **Right:** Correlation matrix between sensory basis network activities for the 32 stimuli. Notably, correlations for stimuli that differ only along task irrelevant dimensions are high. Furthermore, correlations for stimuli that require different actions (type 1 vs. types 2, 3, and 4) are low.

Therefore, we expected that the sensory basis network would develop representations which differentiate well along stimulus dimensions that were task relevant (i.e., stimulus duration and modulation direction) and which are not very discriminative along stimulus dimensions that were not relevant to the task. This effect can be observed in Figure 7. Activities in the basis net are highly similar for stimuli that differ only along dimensions which are not relevant to the task. Furthermore, correlations between stimuli that have a different meaning with respect to the task (i.e., that require different behavioral responses) are low, indicating that the sensory basis network already makes discriminations that are task relevant. This supports strategy learning in the expert networks.

Thus, our model predicts that similar, task-relevant sensory representations should be found in sensory brain regions, e.g., in auditory cortex. We would expect representations of stimuli that belong to the same task category, i.e., that differ only along dimensions that are non-discriminative with respect to the task, to show much higher correlations among each other than with representations of stimuli that belong to a different task category. While this hypothesis still requires further experimental work, there are indications that task-dependent representations exist in sensory cortices (Ohl et al., 2001; Weis et al., 2013).

The assumption of a sensory basis network has another consequence concerning non-discriminative stimulus dimensions. A multi-expert model without sensory basis network predicts that learning speed is independent of whether new stimulus dimensions become task-relevant after a contingency change. As each expert learns the complete sensory-to-motor mapping independent of all others, a newly recruited expert can neither exploit the sensory categories learned by other experts, nor can its learning be impaired by them. In contrast, in architectures with a sensory basis network experts share sensory categories. Thus, it should take longer to learn a new task that requires new sensory categories to be formed because a new stimulus dimension becomes relevant, whereas learning of a task that exploits previously learned sensory categories will be faster. These predictions can be used in future experimental work in order to determine whether human and animal learners do indeed rely on a sensory basis network as predicted in our model.

### 4.6. Possible improvements and extensions

Here, we introduced the concept of a dynamic control network (DCN) that “switches” expert networks once the reward and the expected reward (as calculated by the network) differ strongly. The results presented here indicate that the mechanism of a DCN can explain the ability of the system to recover previously learned responses and quickly regain behavioral performance levels after contingency changes. However, the architecture of the DCN is currently rather simple and leads to several limitations, which should be addressed in future work.

The DCN currently uses hard-wired connections to inhibit the expert networks, similar to the gating proposals in Jacobs et al. (1991a,b); Graybiel (1998). Therefore, it implements a selection mechanism for a fixed number of expert networks. In the simulations reported here, we used a network architecture with three experts. However, in biological networks the number of experts is most likely not constrained in this manner. Thus, it remains to be investigated how the current DCN mechanism can be generalized to an architecture in which experts emerge dynamically during learning. One possible approach would be to have the DCN units inhibit random, overlapping portions of the network instead of clearly separated expert networks (Brosch and Neumann, 2012). In this case, the DCN would steer network dynamics in a more variable way, selecting between different attractor states of the network. This is conceptually similar to recurrent neural networks with a parametric bias (Tani and Ito, 2003; Tani et al., 2004), in which a top-down input signal (the parametric bias) is used to steer network dynamics, making it possible to switch and even combine previously learned behaviors.

In this context it is important to consider that a high number of expert networks may require a different selection mechanism. Currently, our model selects new experts by random search, which would not work reliably for architectures with many experts, as the average number of trials required to find a correct, previously learned strategy grows with the number of expert networks. An alternative to random selection would be to evaluate several expert networks on each trial and to select the most reliable one, similar to Donoso et al. (2014). It remains to be examined how many alternative strategies human and animal learners can learn in parallel, i.e., how many expert networks are required. Donoso et al. (2014) found that a low number of two to three strategies modeled their subjects' performance best, but in their model further inactive strategies could be stored in and retrieved from long-term memory.

An important advantage of our network is that it can learn to switch experts based on the reward prediction error alone and does not require additional sensory contextual information, similar to Bayesian multi-strategy learners (Donoso et al., 2014; Niv et al., 2015). Thus, the proposed model constitutes a first step to understanding neuronal mechanisms for error-based strategy selection. This is crucial in serial reversal learning in which no contextual cues predict contingency changes. Nevertheless, there are also many scenarios in which environmental cues could be exploited to select the appropriate task state. If there are environmental cues indicating a reversal (e.g., ambient lighting changes from green to red) it might be useful to learn a biasing function from the regular input to the winner-take-all units of the DCN. This is conceptually similar to the gating mechanism in Jacobs et al. (1991a). In this way both forms of strategy switching, error-based and context-based, could be incorporated in one model. To what extend the two mechanisms interact requires further investigation, e.g., experiments in which context is indicative of some contingency changes but not of others. It remains to be seen whether participants rely on context and error information equally or disregard one in favor of the other.

Furthermore, the impact of training different expert networks atop a common basis network needs to be further investigated. From a theoretical point of view, this problem is related to recent proposals in the deep learning community trying to train neural networks for multiple objectives, like simultaneous region segmentation and object recognition (Girshick et al., 2014; Ren et al., 2016).

### Conclusion

To summarize, the present contributions are three-fold: First, we demonstrated that the reinforcement learning rule presented in Brosch et al. (2015) is also suitable for behavioral learning as part of a more complex model architecture. Second, we present experimental findings from animal as well as human studies on reversal learning. We show that previously learned stimulus-response mappings can be readopted faster in serial reversal learning. Third, we observed that a standard reinforcement trained neural network cannot account for the faster learning after the second reversal and suggest an extended architecture that captures the results. It consists of a sensory basis network and multiple expert networks, each of which learns a distinct stimulus-response mapping. During each trial, one expert is selected by a dynamic control network to determine the response and to learn based on the reward, while all other experts are inhibited. The model is conceptually similar to other multiple-expert architectures (Jacobs et al., 1991a,b; Frank and Badre, 2012). However, in our model the dynamic control is learned based on the prediction error alone and does not require additional context information. A possible neural substrate for the proposed learning mechanism comprises sensory and association cortices, implementing the basis network for feature extraction, prefrontal cortex as the substrate for the expert networks, and the basal ganglia, realizing the dynamic control network. This hypothesis is consistent with previous proposals about the roles of the prefrontal cortex and basal ganglia in behavioral and reinforcement learning (Graybiel, 1998; O'Reilly and Pauli, 2010; Frank and Badre, 2012) and action selection (Redgrave et al., 1999; Gurney et al., 2001a,b). Thus, we believe that we have identified a mechanism for simultaneous acquisition and dynamic control of multiple action repertoires.

## Author contributions

Manuscript preparation: CJ, TB, HN, MW, AS, FO, ML, AB. Designing the model: CJ, TB, HN. Simulations: CJ, TB, ML, AS. Human experiments: ML, AB. Gerbil experiments: AS, FO, MW.

### Conflict of interest statement

The authors declare that the research was conducted in the absence of any commercial or financial relationships that could be construed as a potential conflict of interest.

## Footnotes

^1^The reduction in the number of units being employed at each processing stage, or model area higher up in the hierarchy, roughly corresponds with the pyramidal structure of sensory networks in a series of areas (Uhr, 1972).
